# Tracing Back Clinical *Campylobacter jejuni* in the Northwest of Italy and Assessing Their Potential Source

**DOI:** 10.3389/fmicb.2016.00887

**Published:** 2016-06-13

**Authors:** Elisabetta Di Giannatale, Giuliano Garofolo, Alessandra Alessiani, Guido Di Donato, Luca Candeloro, Walter Vencia, Lucia Decastelli, Francesca Marotta

**Affiliations:** ^1^National Reference Laboratory for Campylobacter, Istituto Zooprofilattico Sperimentale dell’Abruzzo e del Molise “G.Caporale”Teramo, Italy; ^2^Department of Statistics and GIS, Istituto Zooprofilattico Sperimentale dell’Abruzzo e del Molise “G.Caporale”Teramo, Italy; ^3^Food Hygiene and Safety Department, Istituto Zooprofilattico Sperimentale del Piemonte, Liguria e Valle d’AostaTorino, Italy

**Keywords:** *Campylobacter*, PFGE, population genetic approach, epidemiological investigations, MLST genotyping

## Abstract

Food-borne campylobacteriosis is caused mainly by the handling or consumption of undercooked chicken meat or by the ingestion of contaminated raw milk. Knowledge about the contributions of different food sources to gastrointestinal disease is fundamental to prioritize food safety interventions and to establish proper control strategies. Assessing the genetic diversity among *Campylobacter* species is essential to our understanding of their epidemiology and population structure. We molecularly characterized 56 *Campylobacter jejuni* isolates (31 from patients hospitalized with gastroenteritis, 17 from raw milk samples, and 8 from chicken samples) using multilocus sequence typing (MLST) and pulsed-field gel electrophoresis (PFGE) in order to trace the source of the disease. We also used a population genetic approach to investigate the source of the human cases from six different reservoirs of infection. MLST identified 25 different sequence types and 11 clonal complexes (CCs) (21, 658, 206, 353, 443, 48, 61, 257, 1332, 354, 574) and these included several alleles not cited previously in the PubMLST international database. The most prevalent CCs were 21, 206, and 354. PFGE showed 34 pulsotypes divided between 28 different clusters. At the fine scale, by means of PFGE and MLST, only two human cases were linked to raw milk, while one case was linked to chicken meat. The investigation revealed the presence of several genotypes among the human isolates, which probably suggests multiple foci for the infections. Finally, the source attribution model we used revealed that most cases were attributed to chicken (69.75%) as the main reservoir in Italy, followed to a lesser extent by the following sources: cattle (8.25%); environment (6.28%); wild bird (7.37%); small ruminant (5.35%), and pork (2.98%). This study confirms the importance of correlating epidemiological investigations with molecular epidemiological data to better understand the dynamics of infection.

## Introduction

Human campylobacteriosis in the European Union (EFSA, 2014) continues to be the most commonly reported zoonosis, and with 214,268 confirmed cases, this disease has considerable socio-economic impact. The *Campylobacter* species most commonly associated with human infections are *Campylobacter jejuni* followed by *C. coli* and *C. lari*, but other species, including the non-thermophilic *C. fetus*, also occasionally cause human diseases (EFSA, 2014; Rodrigues et al., 2015). In Italy, 1252 cases of human campylobacteriosis were reported in 2014 (EFSA and ECDC, 2015). However, the data are likely to grossly underestimate the real number of cases (EFSA and ECDC, 2015) because the Italian reporting system for human infectious illnesses does not differentiate between gastroenteritis caused by *Campylobacter* and gastroenteritis caused by the other agents listed in the National Legislation in Italy (Calistri and Giovannini, 2008). Therefore, campylobacteriosis is not a statutory notification illness and the only data available on these infections are those reported voluntarily by Enter-Net, the international network for the surveillance of human gastrointestinal infections.

Generally, campylobacteriosis infections are self-limiting and only last for a few days. However, post-infection complications or extra-intestinal infections such as reactive arthritis and neurological manifestations can also arise (Haddad et al., 2010). Outbreaks caused by thermophilic *Campylobacter* species are most commonly connected with dairy and poultry products, as well as *Campylobacter*-contaminated food and untreated water (Meldrum et al., 2005; EFSA, 2008, 2014; Taylor et al., 2013). Unpasteurized or inadequately pasteurized cow’s milk has been implicated as the sources of infection in some outbreaks (Schildt et al., 2006; Heuvelink et al., 2009), and recently, the European Union summary report on food-borne disease outbreaks confirmed the importance of milk as a source of human campylobacteriosis (EFSA, 2013).

In a recent survey conducted in Northern Italy, *C. jejuni* was detected in 12% of the bulk tank milk samples that were examined (Bianchini et al., 2014), and some disease outbreaks have been reported in the same Italian regions (two in Emilia Romagna, one in Veneto, and one in Marche) following the consumption of raw milk from self-service automatic vending machines (Amato et al., 2007; Arrigoni et al., 2009; Petruzzelli et al., 2011). Despite no official data existing for the incidence of campylobacteriosis associated with raw milk consumption, quantitative risk assessment modeling has estimated that the worst possible scenario for human infections with *C. jejuni* linked to the consumption of raw milk in Italy would exceed 300,000 cases per year (Giacometti et al., 2015).

The high genome diversity and plasticity within the *Campylobacter* genus hampers the surveillance and the outbreak detection. Nevertheless, multilocus sequence typing (MLST) and pulsed-field gel electrophoresis (PFGE) are important and well-established tools that can be used to elucidate the epidemiology of *Campylobacter* cases. MLST is the most common genotyping method successfully applied in population genetic models for clarify the reservoirs of infection, while PFGE is a well standardized technique very useful in localized outbreak investigation (Dingle et al., 2001; Fitzgerald et al., 2001; Sheppard et al., 2009).

The objective of this study was to undertake an epidemio logical investigation of *Campylobacter* infections in 31 patients hospitalized in the summer of 2012 from the Piemonte region in Northern west Italy. Source investigation using MLST and PFGE comprised *C. jejuni* isolates from raw bovine milk and chicken meat. A polymorphism in the *hip0* gene was also identified in some *Campylobacter* strains from raw bovine milk, and new PCR primer set for PCR identification were designed and successfully implemented in this study.

## Materials and Methods

### *C. jejuni* Isolates

A total of 31 cases of campylobacteriosis were registered in August, 2012 in the local health district of Turin, Northern Italy. A temporal and spatial proximity was suspected because of a cluster of infections; consequently, active surveillance was carried on suspicious retail food items. The monitoring found 17 raw milk samples from vending machines and 8 chicken meat samples from a retail market were positive for *Campylobacter*. Here, the 56 *C. jejuni* isolates we investigated were cultured on Columbia blood agar and incubated at 42°C for 48 h in a microaerobic atmosphere.

### DNA Isolation

Genomic DNA was extracted using an UltraClean Microbial DNA Isolation Kit (MO BIO Laboratories, CA, USA) according to the manufacturer’s instructions. The DNA concentration was quantified using a Nanodrop spectrophotometer (Nanodrop Technologies, Italy).

### Strain Identification

The isolates were typed using a PCR method described previously (Marshall et al., 1999; Di Giannatale et al., 2014).

Six strains isolated from raw milk were non-typable using the PCR method described above. Nevertheless, the strains were determined to be *C. jejuni* by 16S-rRNA gene sequencing (Zhou et al., 1997). Furthermore, a new PCR protocol targeting the *hip0* gene was developed to check for miss-typed isolates. The sequences of the primers, which were designed against the regions upstream and downstream of the original amplification sites, were as follows: P3Fs: 5′-GGAAAAACAGGCGTTGTGGGGG-3′ and P3Rs: 5′-CCGAAGAAGCCATCATCGCACC-3′; P3Fi: 5′-CCTGCTTGAAGAGGGTTTGGGTGG-3′ and P3Ri: 5′-TGCAACCTCACTAGCAAAATCCACA-3′. The PCR was perfor med using Master Mix 2X (Promega, Italy), the final concentration of the primers was 1 μM, the annealing temperature was 55°C, and the procedure involved 35 cycles in a thermal cycler 9700 Applied Biosystems (Applied Biosystems, Foster City, CA, USA). Sequencing of the amplified fragment was performed using an ABI PRISM BigDye Terminator 3.1 Cycle Sequencing Kit (Applied Biosystems, Foster City, CA, USA) according to the manufacturer’s instructions, and the sequences were analyzed with an ABI PRISM 3500 Genetic Analyzer (Applied Biosystems, Foster City, CA, USA).

### PFGE

Pulsed-field gel electrophoresis was conducted according to the instructions from the 2009 USA PulseNet protocol for *Campylobacter* (CDC, 2009). The isolates previously identified by PCR were grown on Columbia agar (48 h at 42°C) in a microaerophilic atmosphere and embedded in agarose blocks (Seakem Gold agarose, Lonza, Rockland, MD, USA). Following DNA purification, 1 mm of agar plugs slices was digested 18 h with SmaI restriction enzyme (Promega, Milan, Italy) and the DNA fragments were separated by PFGE (Chef Mapper II, Biorad Laboratories, Hercules, CA, USA) in 1% agarose gel (Seakem Gold agarose, Lonza).

Salmonella Braenderup H9812, digested with *Xba*I enzyme (Promega, Milan, Italy), was used as the standard molecular weight marker. The gel was stained with SYBR Safe DNA gel stain (Invitrogen, Cergy Pontoise, France) and photographed on a UV transilluminator (Alpha Innotech Corporation, San Leandro, CA, USA). The image analysis was performed using Bionumerics program v. 6.6 (Applied Maths NV, Sint-Martens-Latem, Belgium). The similarity analysis was carried out using the Dice coefficient (position tolerance, 1%). The unweighted pair group mathematical average was used to cluster patterns. Isolates with <90% similarity were clustered as separate pulsotypes.

### MLST Analysis

Multilocus sequence typing was performed as reported by Dingle et al. (2001) for all *C. jejuni* isolates. MLST amplified a segment of seven housekeeping genes: *aspA* (aspartase, 477 bp), *glnA* (glutamine synthase, 477 bp), *gltA* (citrate synthase, 402 bp), *glyA* (serine hydroxyl methyl transferase, 507 bp), *pgm* (phosphor glucomutase, 498 bp), *tkt* (transketolase, 459 bp), and *uncA* (ATP synthase, alpha subunit, 489 bp) to yield a total composite sequence length (all seven loci) of 3309 bp. PCRs and sequencing reactions were carried out according to the guidelines on the *Campylobacter* MLST website. Briefly, purified PCR products were sequenced using an ABI PRISM BigDye^®^ Terminator 3.1 Cycle Sequencing Kit (Applied Biosystems, Foster City, CA, USA) according to the manufacturer’s instructions and then analyzed by the ABI PRISM 3500 Genetic Analyzer (Applied Biosystems, Foster City, CA, USA). The alleles, STs, and CCs were identified using the MLST database^1^ Novel alleles were submitted to the PubMLST *C. jejuni/C. coli* database curators for number assignment.

### Source Attribution Analysis

According to a previous study conducted in the Netherlands (Mughini Gras et al., 2012), *Campylobacter* isolates from humans were found to share significant similar ST frequency distribution with those from Europe (the Netherlands, the UK, and Switzerland), but were genetically dissimilar from abroad isolates (from the USA and New Zealand). We have assumed this situation is also true for Italy and the details in our reference database included 558 *C. jejuni* strains from previously published Italian data; Piccirillo et al., 2014) and from our previous survey (Marotta et al., 2015), as well as the 6854 *C. jejuni* from European countries accessible in PubMLST^2^

The reservoir data we identified were pooled and arranged in six groups: (i) chicken, (ii) cattle, (iii) environment, (iv) wild bird, (v) small ruminant, and (vi) pork. Environmental strains comprised those from water, sand, and soil. The population genetics approach used for attributing human campylobacteriosis isolates to the six putative reservoirs was the asymmetric island model described by Wilson et al. (2008). This model estimates the recombination rates within the reservoirs, between the reservoirs and from each reservoir in the human population to estimate the posterior distribution used to infer the fraction of human cases attributable to each source.

### Diversity Index and Evaluation of the Combined Typing Methods

The discriminatory ability of the two typing systems (PFGE and MLST) was measured according to Simpson’s diversity index. The concordance of the methods was determined by calculating the adjusted Rand and Wallace coefficients. The first coefficient is used to evaluate the extent of agreement between two typing methods, and the second method is an estimate of how much new information is obtained from a typing method in comparison with another one (Behringer et al., 2011). The adjusted Rand and Wallace coefficients were used to calculate the index from the Online Tool for Quantitative Assessment of Classification Agreement^3^

## Results

**FIGURE 1:**
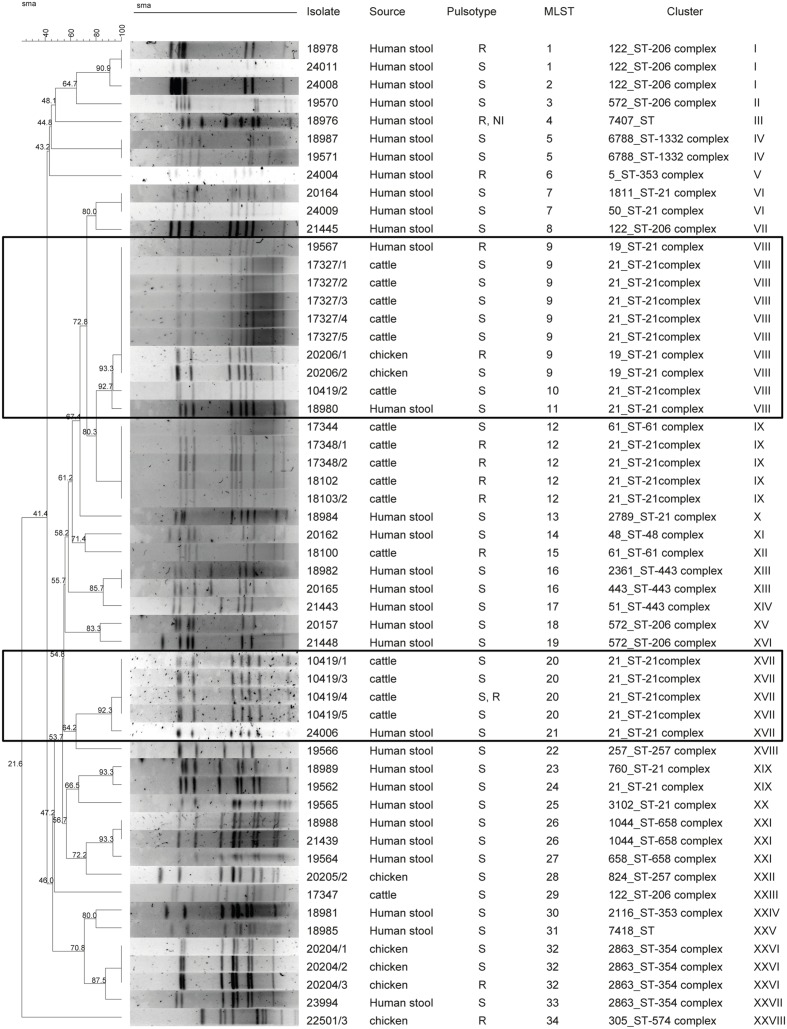
**Comparison of pulsed-field gel electrophoresis (PFGE) and multilocus sequence typing (MLST) among *Campylobacter jejuni* isolates from different sources**.

**FIGURE 2:**
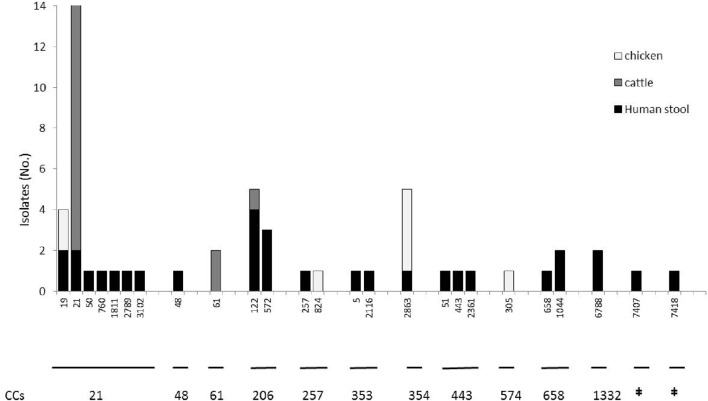
**Frequency distribution of the *C. jejuni* clonal complexes (CCs) isolated from chickens, human stool and cattle samples (*n* = 56) in the Piemonte region of Italy.** STs belonging to the CCs listed below are reported on the *x* axis, while the *y* axis represents the number of isolates. ^‡^No clonal complex applicable.

A total of 55 *C. jejuni* isolates were typed with PFGE using *Sma*I restriction enzyme. One chicken isolate could not be typed. Thirty-four different pulsotypes were obtained from the isolates typed (**Figure 1**). The cluster analysis distinguished the isolates according to 28 main clusters, with a similarity of 90% (**Figure 1**). Among these, only two clusters included non-human sources and three clinical isolates. Cluster VIII contained two human isolates, six raw milk isolates and two chicken meat isolates, and cluster XVII, contained one human and four raw milk isolates (**Figure 1**). MLST typing for the 56 isolates identified 25 STs featured in 11 clonal CCs (**Figure 2**). The most prevalent CCs were 21, 206, and 354. CC21 was the largest one shared by humans, cattle, and chickens, while CC206 and CC354 included human and cattle isolates or human and chicken isolates, respectively. At the fine scale, in three cases human isolates were linked to chicken sources sharing the STs 19 and 2863, and in six cases the human isolates were linked to raw milk sources sharing STs 122 and 21. MLST revealed the presence of 18 STs that were not attributable to any of the sources we investigated, including three new STs (STs 7407, 7418, and 6788), probably suggesting multiple foci of infections (**Figure 2**, **Table 1**).

**Table 1:** Genetic diversity and frequency distribution of 56 *Campylobacter jejuni* strain isolates from human stool, chicken, and cattle sources.

Clonal complex	ST	Multilocus sequence typing (MLST) allelic profile							Pulsed-field gel electrophoresis (PFGE) profile^a^	Source and number of isolates
				
		aspA	glnA	gltA	glyA	pgm	tkt	uncA	SmaI cluster	
21	21	2	1	1	3	2	1	5	**VIII–XVII**	Human stool (3)
										Cattle (14)
	19	2	1	5	3	2	1	5	**VIII**	Human stool (1)
										Chicken (2)
	1811	2	4	12	3	2	1	5	VI	Human stool (1)
	2789	2	1	12	3	2	3	5	X	Human stool (1)
	3102	2	84	12	3	11	1	5	XX	Human stool (1)
	50	2	1	12	3	2	1	5	VI	Human stool (1)
	760	2	1	52	3	2	100	5	XIX	Human stool (1)
658	658	2	4	2	4	19	3	6	XXI	Human stool (1)
	1044	2	10	2	4	19	3	6	XXI	Human stool (2)
206	122	6	4	5	2	2	1	5	XXIII	Cattle (1)
									I–II	Human stool (4)
	572	62	4	5	2	2	1	5	II–XV–XVI	Human stool (3)
353	2116	7	17	52	10	89	3	6	XXIV	Human stool (1)
	5	7	2	5	2	10	3	6	V	Human stool (1)
443	443	24	17	2	15	23	3	12	XIII	Human stool (1)
	2361	7	254	2	15	23	3	12	XIII	Human stool (1)
	51	7	17	2	15	23	3	12	XIV	Human stool (1)
48	48	2	4	1	2	7	1	5	XI	Human stool (1)
61	61	1	1	4	2	2	6	3	XII–IX	Cattle (2)
257	257	9	2	4	62	4	5	6	XVIII	Human stool (1)
	824	9	2	2	2	11	5	6	XXII	Chicken (1)
^‡^	7407^§^	24	28	2	28	10	1	125	III	Human stool (1)
^‡^	7418^§^	24	17	52	10	89	418	6	XXV	Human stool (1)
1332^§^	6788^§^	2	1	4	28	58	29	58	IV	Human stool (2)
354	2863	198	2	2	2	11	61	6	XXVI	Chicken (4)
									XXVII	Human stool (1)
574	305	9	53	2	10	11	3	3	XXVIII	Chicken (1)


*^a^PFGE profiles for the restriction enzyme *Sma*I. Bold font: the two identified clusters shared among humans and cattle or humans and chicken.*

*^§^ New allelic combination.*

*^‡^No clonal complex applicable.*

Overall, the 31 human samples were assigned to 22 STs belonging to 11 CCs; among them, two clonal complexes have not been assigned to the international database yet. By means of the two techniques employed here and as shown in **Figure 1**, only 3 out of 31 cases were truly traceable back to the two cases linked to raw milk from vending machines, to chicken meat and one human case linked to raw milk.

The Simpson’s index and the confidence intervals for PFGE (0.949, 95% CI = 0.920–0.979) were higher than for MLST (0.897, 95% CI = 0.834–0.962), *p* = 0.044. PFGE is a more discriminatory typing method than MLST; however, when comparing the 95% confidence intervals, it is noteworthy that they overlap. Therefore, we cannot exclude the hypothesis that both methods have similar discriminatory power (at the 95% confidence level).

The concordance of the methods based on the calculation of the adjusted Rand coefficient and 95% CI shows a concordance between MLST and PFGE of 0.32 (95% CI = 0.209–0.437). The concordance based on the Wallace coefficient was also higher for the PFGE–MLST combination (Wallace = 0.505, 95% CI = 0.290–0.714), with respect to the MLST–PFGE combination (Wallace = 0.236, 95% CI = 0.120–0.343), *p* = 0.019.

**FIGURE 3:**
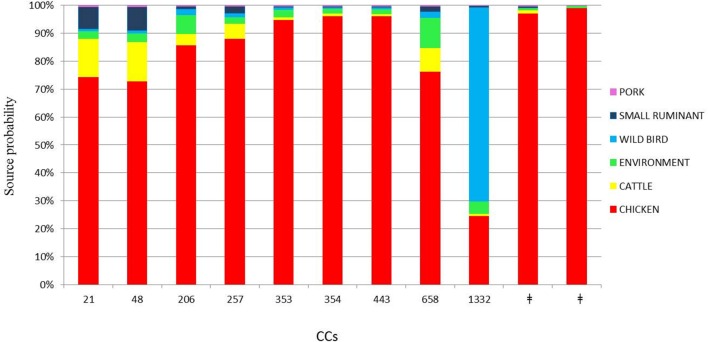
**Attributed probability (%) for the six most represented clonal complexes originating from chicken, cattle, environment, wild bird, small ruminant, and pork sources.** ^‡^No clonal complex applicable.

The attribution analysis revealed that 69.75% of the human cases were predominantly related to chicken (**Figure 3**). Interestingly, only two human cases belonging to CC1332 were predominantly related to wild bird (62.42%, **Figure 3**). In the asymmetric island model, most human cases (69.7%) were attributed to the chicken reservoir (95% CI 47.6–87.5%). Cattle were the second most probable source reservoir (8.2%, 95% CI 0–25.6%) followed by wild birds (7.3% CI 0–19.9%), the environment (6.2% CI 0–22%), small ruminants (5.3% CI 0–17.8%) and pork (2.9% CI 0–10.7%). Although there was a large credibility interval for the chicken source, the model gave the most probable source at a probability of 99.8%.

**FIGURE 4:**
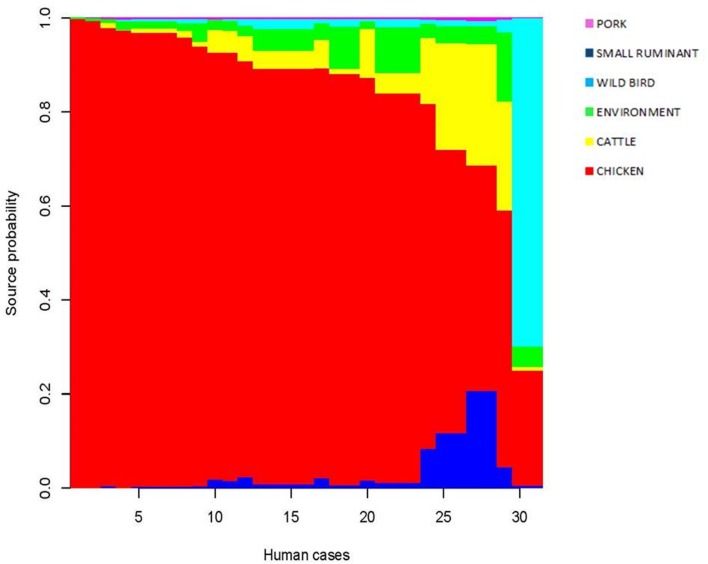
**Posterior probability of the source for each isolate**.

For each patient, the model restituted the posterior probability for the source of infection as shown in **Figure 4**. One dominant color confirmed that chicken is the likely source for the cases under consideration. It is worth noting that, for the generalist CC21 and CC48 regularly isolated from multiple reservoirs, the model showed a minor probability of chicken in favor of ruminants (cattle and small ruminants). CC1332 featured two clinical isolates and resulted with wild birds as the most probable source of infection. Overall, the 31 human cases investigated were probably infected in 29 instances by chicken and in the last two cases by wild birds. A minor probable source was confirmed for pork.

## Discussion

The percentage of human cases with campylobacteriosis ascribed to eating or handling raw poultry differs between countries and studies. Estimates of cases of a food-borne origin range from 30 to 58% (Mullner et al., 2009), and up to 80% can be attributed to the chicken sources as a whole (EFSA, 2011; Mughini Gras et al., 2012). Furthermore, the sources of the notified cases have not been able to be determined, indicating that the real number of food-borne cases is unknown (Meinersmann et al., 2005; EFSA, 2013). It is well known that the presence of *C. jejuni* in bovine raw milk samples and fresh dairy products is increasing (Fernandes et al., 2015). Recent data show that the consumption of undercooked chicken meat and raw milk are the most important sources of human campylobacteriosis in Europe (EFSA, 2014), accounting for hundreds of cases. Outbreaks associated with the consumption of raw or pasteurized milk contaminated with *Campylobacter* were recently recorded in Italy (Giacometti et al., 2012). A quantitative risk assessment focused on one region of Northern Italy evaluated that 1–2% of the population were consumers of raw milk from vending machines and only 57% of users boiled the raw milk before consumption; therefore, the estimated proportion of the people consuming unboiled raw milk is 0.5–0.9% (4548–9096 people; Giacometti et al., 2012).

The present study aimed to investigate an outbreak of campylobacteriosis among humans and to define the infection sources. An active surveillance was undertaken that sampled raw milk from vending machines and chicken meat from a retail market. The PFGE and MLST results suggested that the human cases we studied were not caused by a single-point source, but were likely triggered by multiple foci of infection instead. The evidence supporting this conclusion was the presence of 26 different pulsotypes discovered by PFGE and 22 STs for MLST. The MLST results found nine clinical cases with similar profiles for the raw milk and chicken samples. In particular, ST21 and ST122 were shared among humans and cattle, while ST19 and ST 354 were shared between humans and chickens.

Calculation of the two coefficients (adjusted Rand and Wallace coefficients) and their confidence intervals permits quantification of the congruence between the two different method results (Wallace, 1983; Pinto et al., 2008). In our study, the adjusted Rand coefficient gave low congruence between the different methods. The PFGE analysis showed that isolates with the same MLST profile were partially congruent and sometimes grouped inside different clusters, as was also revealed by the adjusted Rand coefficient between MLST and PFGE of 0.32 (95% CI = 0.209–0.437). However, the Wallace coefficient for PFGE and MLST gave a value of 0.5, which is higher than the 0.2 value from the MLST–PFGE combination, suggesting that PFGE performs better in an outbreak scenario.

Nevertheless, only three cases featured in VIII and XVII groups clustered together with chicken and raw milk samples, a finding in agreement with the MLST results. In contrast, ST 122 and ST 354 did not cluster together in the PFGE profiles, suggesting a possible diverse origin for them. In this study, the clustering concordance of the two molecular techniques is not similar, but we propose that complementation of the results could lead to a higher level of isolate discrimination.

The data suggest that genomic-based techniques like MLST and PFGE can provide useful insight for outbreak investigations. PFGE has been standardized for *Campylobacter* spp., and has been used in localized outbreak investigations (Fitzgerald et al., 2001), while MLST is useful for investigating the DNA sequence diversity via the index of variations in housekeeping genes (Dingle et al., 2001).

Recently, MLST has commonly been employed to increase the analysis resolution of outbreak-associated isolates, leading to quicker and much more accurate source identification, as well as investigating different epidemiological hypotheses.

Our findings revealed three human cases that had similar genetic profiles to chicken and cattle isolates. However, in this study, there were no clues for tracing the remaining isolates investigated and we did not retrieve similar profiles from the source strains isolated during the active surveillance. Therefore, to try to understand the source of infection, we applied a genetic population approach using MLST data from our database and the data from other studies conducted in Europe.

From our analysis, chicken was estimated to be the most dominant source of infection for the human isolates we studied. Using asymmetric island model, the MLST profiles associated with human disease were most similar to those from a chicken source in 69.75% of the cases of *C. jejuni* infection. Ruminants (cattle and small ruminants) contributed far fewer cases of *C. jejuni* infection (13.6%) and the contribution from environment, wild bird, and pig sources was very low (6.28, 7.37, and 2.98%, respectively). This finding is in line with many other studies performed in industrialized countries, even if divergence in the percentage of cases attributable to the chicken source varied among these studies (Wilson et al., 2008; Mullner et al., 2009; Sheppard et al., 2009). Our results should indicate the importance of chicken as the main reservoir of human campylobacteriosis and lend to the suggestion that this disease could be greatly reduced by focusing interventions on chickens. The cases studied are not representative of the Italian epidemiological context but seem to be congruent with other European scenarios.

Notably, inspecting the three cases that were traced back to a potential source revealed that the model estimated chicken as the most probable source of infection for all the cases as well. Contrastingly, our investigation found that one case featured in PFGE cluster VIII and ST 21 was attributable to raw milk instead of chicken. In fact, CC21 is a multi-host lineage shared among different sources, and this can lead to an incorrect assignment in the genetic model. The model calculates the likely source of the infection but cannot rule out completely the minor reservoir as the actual source. In this case, the back-tracing analysis using PFGE and MLST is likely to be the best approach for drawing the correct reconstruction of the food-borne illness. In this scenario, whole-genomeı sequencing (WGS) would probably provide the best fit for the epidemiological analysis, but this approach is not yet ready to be applied routinely because of the lack of standardization of the analysis of the WGS data in the context of the molecular epidemiology of food-borne pathogens.

## Conclusion

The number of clinical isolates, albeit limited to only 56 samples from a restricted area and in a short time window, confirms the high genetic diversity present in *Campylobacter*. Genetic assessment of *Campylobacter* spp. is fundamental to our understanding of its epidemiology. Additional analyses of isolates from various sources will consent major progresses in the knowledge of the epidemiology and population structure of *C. jejuni* in Italy. This study confirms the interest of molecular epidemiology as a powerful support for epidemiological investigations.

## Author Contributions

EG has contributed to the conception and design of the work and wrote the paper. AA performed experiments. FM performed experiments, analyzed data and wrote the paper. GG performed the source attribution analysis, analyzed data and wrote the paper. LC performed the source attribution analysis. GD helped draft the manuscript. WV and LD contributed strains.

## Conflict of Interest Statement

The authors declare that the research was conducted in the absence of any commercial or financial relationships that could be construed as a potential conflict of interest.

## Footnotes

1 http://pubmlst.org/campylobacter/.

2 http://pubmlst.org/.

3 http://darwin.phyloviz.net/ComparingPartitionsv2/index.php?link=Tool.
